# Expert-enhanced machine learning for cardiac arrhythmia classification

**DOI:** 10.1371/journal.pone.0261571

**Published:** 2021-12-23

**Authors:** Sebastian Sager, Felix Bernhardt, Florian Kehrle, Maximilian Merkert, Andreas Potschka, Benjamin Meder, Hugo Katus, Eberhard Scholz

**Affiliations:** 1 Department of Mathematics, Otto-von-Guericke University, Magdeburg, Germany; 2 Informatics for Life, Heidelberg, Germany; 3 Department of Internal Medicine III, University Hospital Heidelberg, Heidelberg, Germany; 4 Institute of Optimization, Technical University Braunschweig, Braunschweig, Germany; 5 Institute of Mathematics, Clausthal University of Technology, Clausthal-Zellerfeld, Germany; 6 German Centre for Cardiovascular Research, Heidelberg, Germany; 7 GRN Gesundheitszentren Rhein-Neckar gGmbH, Schwetzingen, Germany; Ulm University, GERMANY

## Abstract

We propose a new method for the classification task of distinguishing atrial fibrillation (AFib) from regular atrial tachycardias including atrial flutter (AFlu) based on a surface electrocardiogram (ECG). Recently, many approaches for an automatic classification of cardiac arrhythmia were proposed and to our knowledge none of them can distinguish between these two. We discuss reasons why deep learning may not yield satisfactory results for this task. We generate new and clinically interpretable features using mathematical optimization for subsequent use within a machine learning (ML) model. These features are generated from the same input data by solving an additional regression problem with complicated combinatorial substructures. The resultant can be seen as a novel machine learning model that incorporates expert knowledge on the pathophysiology of atrial flutter. Our approach achieves an unprecedented accuracy of 82.84% and an area under the receiver operating characteristic (ROC) curve of 0.9, which classifies as “excellent” according to the classification indicator of diagnostic tests. One additional advantage of our approach is the inherent interpretability of the classification results. Our features give insight into a possibly occurring multilevel atrioventricular blocking mechanism, which may improve treatment decisions beyond the classification itself. Our research ideally complements existing textbook cardiac arrhythmia classification methods, which cannot provide a classification for the important case of AFib↔AFlu. The main contribution is the successful use of a novel mathematical model for multilevel atrioventricular block and optimization-driven inverse simulation to enhance machine learning for classification of the arguably most difficult cases in cardiac arrhythmia. A tailored Branch-and-Bound algorithm was implemented for the domain knowledge part, while standard algorithms such as Adam could be used for training.

## Introduction

### Automatic classification of cardiac arrhythmias

The recent success of ML algorithms to classify cardiac arrhythmias is impressive [1]. However, the authors of this survey state: *“A known limitation of current ML methods is that it is challenging to understand the rationale behind their results. The algorithms are not able to provide explanations for the pathophysiological basis of classification outcomes, as they are unable to reveal the functional dependencies between data inputs and classes.”* We agree with this point of view. For example, it is usually not clear if the classification results [2–5] were due to heart rate variability, the particular shape of the electrocardiogram (ECG) curve (including low voltage flutter waves that correspond to atrial polarizations), or a mix of both. Wavelets have been used to extract features automatically [6], but this approach is limited to easy classification cases and does not directly provide physiologically interpretable features. Usually, parameters such as atrial cycle length are not provided, although they may be relevant for treatment decisions [7].

Moreover, none of the surveyed studies addressed the especially difficult case of atrial fibrillation (AFib) versus regular atrial arrhythmias including atrial flutter and focal atrial tachycardias with irregular ventricular response (AFlu), summarized as AFib↔AFlu hereafter. It is either completely omitted as in [6], which focuses on the classification classes normal beat, left bundle branch block beat, right bundle branch block beat, atrial premature beat, paced beat, and premature ventricular contraction, or both physiological cases are grouped together in deep learning (*“The atrial fibrillation class combined atrial fibrillation and atrial flutter”* [3]) and algorithms based on heart rate variability for smartwatches [8]. Studies that explicitly address “detection of AFib” in the title [9–11] can only detect the grouped class of irregular ventricular response which may either be due to AFib or to AFlu. The reason for this is that the special case AFib↔AFlu is difficult. The typically available data, a surface ECG or a time series of heart beats, look very similar in both cases to most laymen, physicians, and computerized algorithms alike. High rates of misdiagnosis and possible causes have been reported [12–14]. This is concerning, as different treatments (often antiarrhythmics in AFib versus a highly successful ablation therapy in AFlu) are implied by the diagnosis [15]. Diagnosing atypical forms of AFlu is becoming increasingly important in clinical practice due to complications of left atrial ablation procedures [16]. See Scholz et al. (*“Discriminating atrial flutter from atrial fibrillation using a multilevel model of atrioventricular conduction”*) [17] for a more detailed discussion. The poor quality of expert opinion due to the difficult discrimination poses a challenge to automated classification by supervised ML, which often uses it for labeling training samples [3–5]. We used an expert analysis based on intracardiac measurements, which is only available with invasive procedures, as our gold standard.

Interestingly, the case AFib↔AFlu seems to be difficult for deep learning approaches. As stated before, the differentiation between AFib and AFlu has been avoided in Hannun et al. (*“Cardiologist-level arrhythmia detection and classification in ambulatory electrocardiograms using a deep neural network”*) [3], where a deep convolutional net with 34 layers was trained using 91232 singe-lead ECGs. Moreover, our results show poor performance of neural-network-based approaches. We conjecture that this is due to the non-continuous nature of the underlying process, which contrasts to the approximation properties of deep neural networks and the relatively small size of the training set.

### Complementing previous work in automatic arrhythmia classification

**Fig 1:**
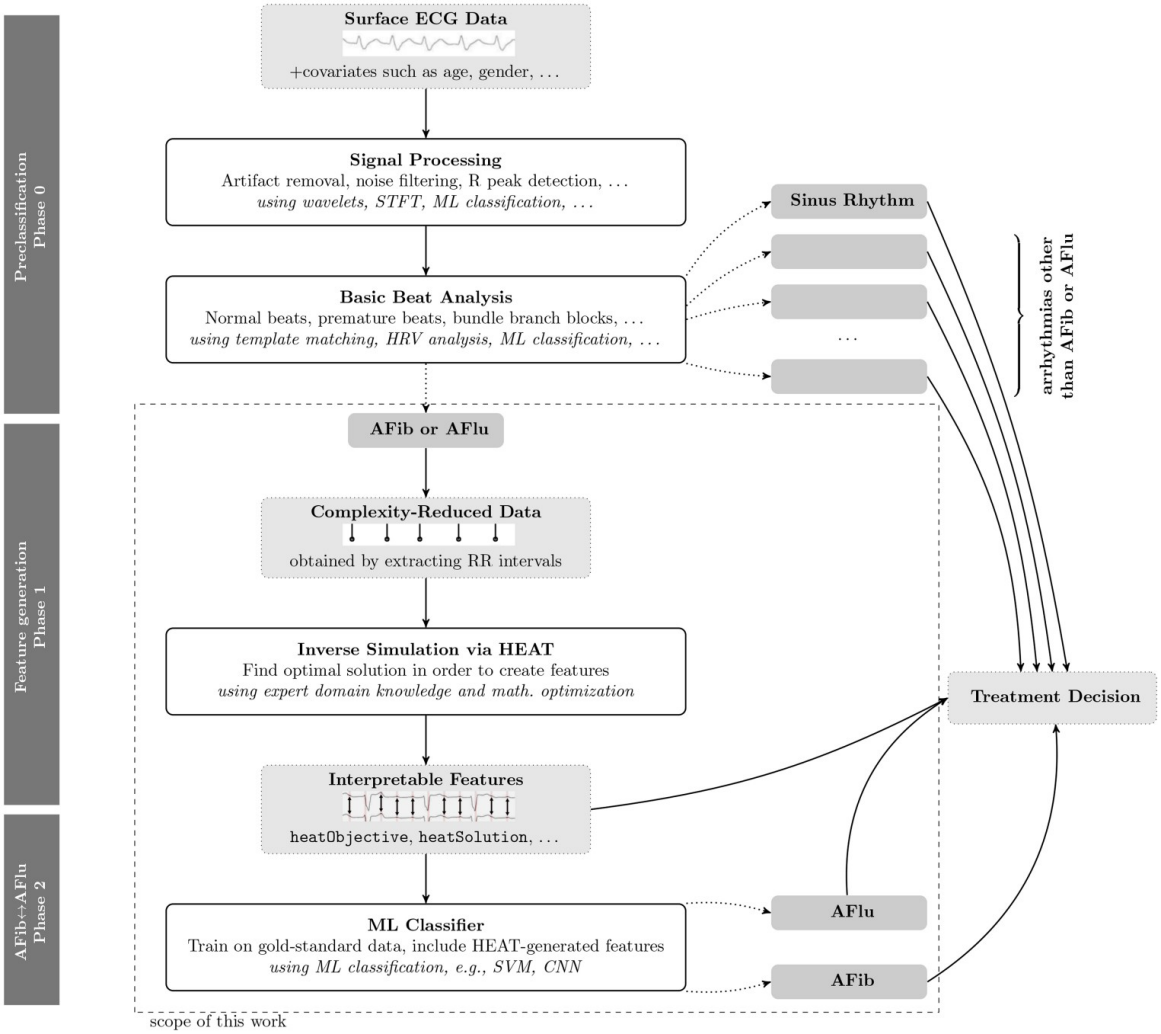
Visualization of our workflow from surface ECG to decision support for treatment. We focus on phases 1 (generation of physiologically interpretable features) and 2 (using them for AFib↔AFlu classification), thus assuming a pre-classification of all samples.

Fig 1 visualizes our workflow. Deep learning (DL) can robustly distinguish samples of either AFib or AFlu from sinus rhythm and twelve cardiac arrhythmias [3] with high accuracy. Other studies achieved similar results [6, 9–11]. For a survey on general ECG-based automatic arrhythmia classification, see Luz et al. (*“ECG-based heartbeat classification for arrhythmia detection: A survey”*) [18].

As a reliable pre-classification (Phase 0) can thus be achieved, we focus here on Phase 1 (generation of physiologically interpretable features) and Phase 2 (using them for AFib↔AFlu classification). In the following, we assume that it has been verified that only either AFib or AFlu is present, which is also true for our gold standard data set (expert classification of intracardiac measurements that are only available after invasive procedures).

We propose to extend and complement the mentioned approaches with generated features based on a pathophysiological rationale allowing classification of AFib↔AFlu. Thus, our approach is not an alternative to previous work of automatic classification, but is rather complementary to it. In previous works, neural networks were trained with genetic algorithms [6] or with tailored stochastic gradient methods [3]. Our approach differs as it uses optimization in two different phases. In Phase 1, features are generated solving mixed-integer optimization problems. In Phase 2, an automatic classification is calculated using optimization. This approach is very modular and any classification algorithm can be applied in Phase 2.

### Feature generation and hybrid modeling

Feature construction has a long history, with early work dating back to the 1960s [19]. Since then, there has been a plethora of feature generation methods, such as polynomial [20], discretization [21, 22], normalization [23], or grouping operations involving min, max, averaging, etc. The current state-of-the-art in feature construction methods suffer from three main drawbacks: exponential explosion of the feature space, difficulty to embed domain knowledge, and loss of interpretability. While the first drawback can be mitigated by feature selection methods, which themselves can be based on machine learning technology [24], the difficulty to embed domain knowledge and to interpret the automatically generated and selected features still remains. Our proposed feature generation overcomes the three drawbacks. Because it is based on the idea to embed domain knowledge (distilled into a mathematical optimization model), the generated features provide insightful interpretation to experienced medical practitioners. in addition, exponential explosion of the feature set is not an issue because only a few additional real-valued features need to be added.

As our feature generation procedure uses only the input data (RR interval times) and is based on optimization, the whole procedure can be seen as a completely novel machine learning model, with a nested hybrid structure. The outer level contains a classical ML part such as a support vector machine (SVM), and at the inner level an inverse simulation domain knowledge model. The optimization on the outer level interacts with the results of the optimization at the inner level.

Combining machine learning models with domain knowledge is an active and promising field of research, e.g., [25, 26]. A survey on how first principle models can be combined in different ways with generic machine learning models is given in Bikmukhametov et al. (*“Combining machine learning and process engineering physics towards enhanced accuracy and explainability of data-driven models”*) [27] in the context of process engineering systems. One way is to replace uncertain parts in differential equations with neural nets using the concept of universal differential equations [28]. ML can also be applied to make the solution of differential equations more efficient [29]. The alternative is to develop and use physics-informed or biology-informed machine learning approaches [30–34]. The general idea is to design ML models such that important physical properties like conservation laws are automatically fulfilled. This promising line of research is often linked to the simulation of complex flows. A physics-informed neural network was applied to noisy clinical data in Kissas et al. (*“Machine learning in cardiovascular flows modeling: Predicting arterial blood pressure from non-invasive 4D flow MRI data using physics-informed neural networks”*) [35]. Here, arterial pressure was predicted from MRI data of blood velocity and wall displacement. Common results of these studies show that by combining physics-based and machine learning models it is possible to improve the performance of the purely black-box ML models making them more transparent and interpretable.

The mathematical model develop and applied in this study can be seen as a simplification of first-principle models for electrical conductivity in the heart, such as the Hodgkin–Huxley equations [36]. In this sense, our approach can also be interpreted as a biology-informed machine learning approach. See Villaverde et al. (*“Structural Properties of Dynamic Systems Biology Models: Identifiability, Reachability, and Initial Conditions”*) [37] for a survey of systems biology models and important properties.

### Summary of our approach

The most important building block in Phase 1 is the inclusion of medical expert knowledge. It was unclear for a long time which role the atrioventricular (AV) node played in the transfer of fast but regular activations of the atrial chambers into irregular activations of the ventricular chambers. As Douglas P. Zipes stated in 2000, the AV node is still *“a riddle wrapped in a mystery inside an enigma”* [38]. Key to solving this riddle is the idea of a multilevel AV block (MAVB) [39–43]. The tedious procedure of manually adjusting possible MAVB combinations has been successfully automated in the algorithm HEAT (Heidelberg Electrocardiogram Analysis Tool, [17]). The underlying hypothesis is that fast but regular activations of the atrial chambers result in irregular responses of the ventricles because of a multilevel succession of simple blocks of *Type I* or *II*. We considered atrial cycle length, blocktype, a vector of blocktype-specific internal offset counters and conduction constants as optimization variables. For different values of these variables, forward simulation of ventricular responses (RR interval lengths) is possible, which can be compared to given RR measurements. A penalization of the difference in an appropriate metric gives a suitable objective function. In an inverse simulation, HEAT can calculate optimal solutions resulting in the smallest deviations for each training sample. The combination of a mathematical model and optimization algorithm could be seen as an interpretable expert system. The basic idea of using a mathematical model and inverse simulation for AFib↔AFlu classification has been published before in [17]. We report a significantly matured approach with a larger (4×) data set which allows for a systematic cross-validation, an improved mathematical model of MAVB with a better pathophysiological interpretation, a computational speed up to 5000×, and an increased accuracy (the area under the ROC of 0.9 in [17] was not cross-validated). Most importantly, for the first time we use HEAT for multi-dimensional ML feature generation and show the advantages of using clinical domain knowledge. The general approach to use domain knowledge plus combinatorial optimization for feature generation may overcome intrinsic approximation limits of deep learning for difficult-to-label and non-smooth systems that often occur in medicine and biology [44–47].

### Structure of this paper

The paper is structured per PLOS One guidelines. In Section Methods we describe our machine learning approach and data. In particular, we explain a mathematical model that is used as domain knowledge to describe AFlu and derived features. In Section Results we present numerical results showing that the proposed approach reaches an unprecedented accuracy, while a direct use of neural networks perform poorly on the data. In Section Discussion we discuss these results in several directions: approximation properties of machine learning as a possible explanation, accuracy and impact, interpretability, and transfer to other clinical domains. Concluding remarks are given in Section Conclusions.

## Methods

### Multilevel atrioventricular block (MAVB)

We developed a mathematical model for MAVB based on the following rationale. In physiology, *refractoriness* specifies the time period in which a cell is incapable of repeating a certain action. Applied to any component in the cardiac conduction system, the *absolute refractory period* (ARP) describes the duration in which a cell cannot be stimulated under any circumstances. The *relative refractory period* (RRP) describes the duration in which the cells can be stimulated under certain conditions, but may react with a modified conduction [48]. Depending on incoming signal and RRP, a block ratio of *n* + 1: *n* can occur, where *n*+ 1 is the number of incoming signals, and *n* the number of conducted signals. This ratio may vary due to changes in cell fatigue or in the frequency of the incoming signals, even on short time horizons. For larger values of *n* the conduction times may change as well.

**Fig 2:**
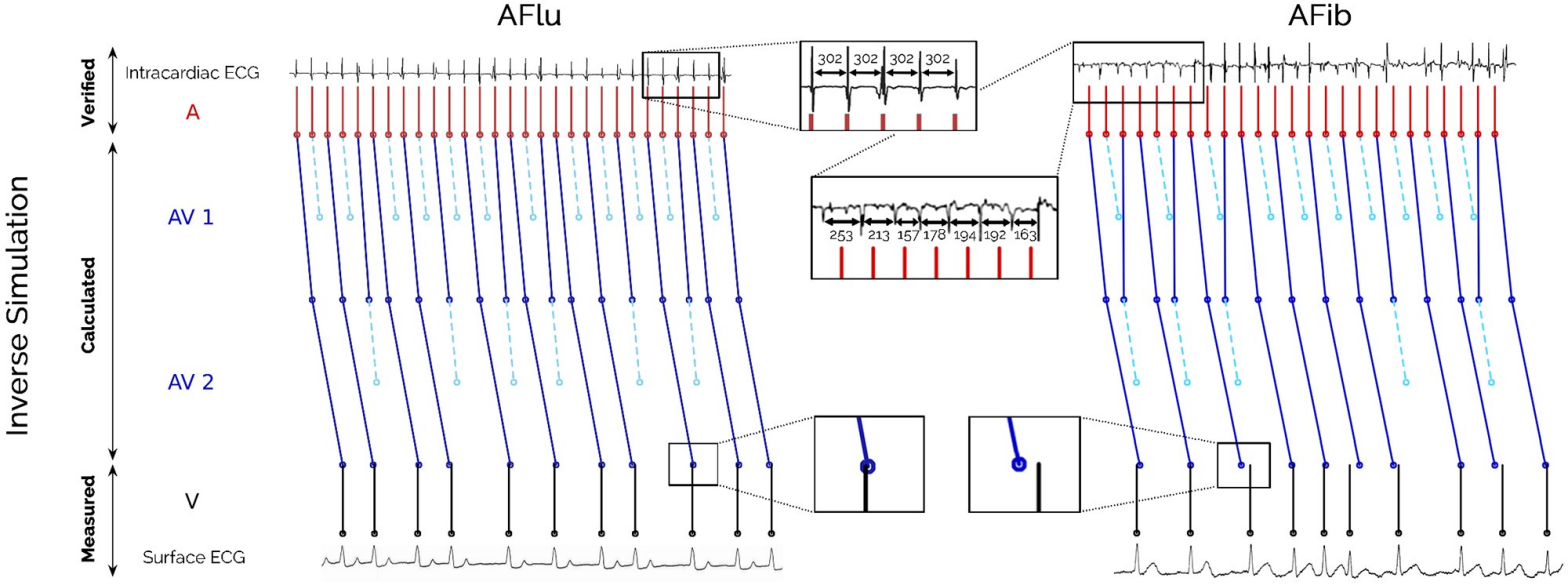
Visualization of our inverse simulation approach applied to samples of atrial flutter (AFlu, left, regular intracardiac measurement) versus atrial fibrillation (AFib, right, irregular intracardiac measurement) based on the surface electrocardiogram (ECG, bottom). In this example, a two-level atrioventricular (AV) block was calculated for both samples.

Reviewing the physiology of the AV node, we considered it as a series of cell compounds in which a signal may potentially be blocked. Hence, the outgoing signal of block level I becomes the incoming signal of block level II (see Fig 2).

Classifying atrial flutter with irregular ventricular response (AFlu, left) versus atrial fibrillation (AFib, right) based on the surface electrocardiogram (ECG, bottom) is difficult for experts and algorithms. If intracardiac measurements were available after invasive procedures, like in our data set, the classification would be easier, allowing the measurements to be used as a gold standard for training of machine learning models and for a-posteriori analysis. The input data of the feature generation, the measured ventricular (V) signals (rawRR), were extracted from the surface ECG. For both samples, a two-level atrioventricular (AV) block was calculated such that the model parameter Δ*a*, the cycle length in the atrial chambers (A), is regular and the forward simulation in V is close to rawRR. We hypothesized that a small deviation (left) can be interpreted as a high likelihood for regular behavior (AFlu), and a large deviation (right) for chaotic behavior which cannot be explained well by the model (AFib). Comparing bottom zooms in Fig 2, cf. Scholz2014, it visually confirmes that for AFlu the calculated Δ*a* corresponds well to the intracardiac measurements.

**Fig 3:**
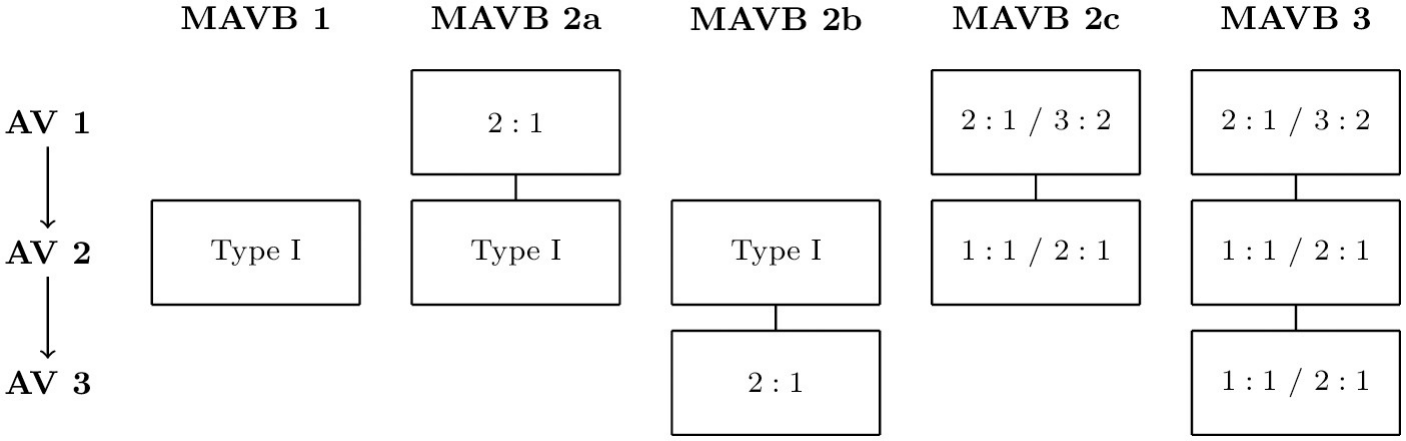
The five considered blocktypes, having up to three multilevel atrioventricular block (MAVB) levels.

This theoretical concept allows to combine different blocking ratios *n* + 1: *n* on an unlimited number of levels. Possibly varying and linearly changing conduction times due to RRP are denoted as *Type I*. Sensibly, the number of possible combinations should be limited to avoid overfitting, reduce computational time, and stay close to clinical observations. We restricted our MAVB model to the five combinations shown in Fig 3 with a maximum of three block levels, consistent with cases described in recent publications.

The resulting mathematical model is a combination of most different classical and advanced block types, particularly, typical Type I block [49–51], atypical Type I block [50, 52], the special cases of 2:1 and 3:2 Type I blocks, Type II block [53–56], advanced second-degree AV Block [57, 58], and MAVB [39–43]. Invoking Occam’s razor, this unified model also allows an efficient calculation of the most likely block for given RR data.

### HEAT

For the inverse simulation optimization problem we considered optimization variables *x* = (Δ*a*, *bt*, *oc*), where Δ*a* is the atrial cycle length, *bt* the blocktype, and *oc* a vector of auxiliary variables representing blocktype-specific internal offset counters and conduction constants. Internally, time points *t*_*ij*_ are calculated and denote, when the signal originating from signal *j* in the atrium reaches level *i*. Due to the assumed regularity in the atrium we have

$$\begin{array}{c} t_{0j}≔t_{s}+j\Delta a \end{array}$$

with an unknown offset *t*_*s*_. On levels 1, 2, and 3 the equations for *t*_*ij*_ depend on the particular blocking type *bt*, and hence more complicated case differentiations: if the signal can be conducted,

$$\begin{array}{c} t_{ij}≔t_{i-1,j}+f\left(oc\right) \end{array}$$

with a linear function *f* depending on parameters *oc*, otherwise it will be blocked and can not be considered in the objective function. Details can be found in the PhD thesis [59] and in the survey paper [60]. The objective function is denoted by *F*_*i*_ where *F*_*i*_(*x*) measures the deviation of the resulting forward simulation based on *x* from the actual RR data sample *i* in the Euclidean norm.

With the help of the software package, HEAT, we calculated for all training samples *i* optimal solutions $x_{i}^{*}$, particular values for $\Delta a_{i}^{*}$, $bt_{i}^{*}$, and $oc_{i}^{*}$ that resulted in the smallest objective function value

$$\begin{array}{c} F_{i}\left(x_{i}^{*}\right)=\underset{x\in X}{\text{min}}\;F_{i}\left(x\right). \end{array}$$

Here, X denotes the feasible set for (Δ*a*,*bt*, *oc*) with lower and upper bounds for (Δ*a*, *oc*) and five most clinically observed blocktypes of MAVB (see Fig 3). The bounds on the atrial cycle length Δ*a* were determined using physiological observations [48] (between 175ms and 400ms) and dependent on the blocktype *bt* and the input RR data. The algorithm is based on an intelligent enumeration (comparable to Dynamic Programming or Branch & Bound) of all possible solutions, assuming a time grid of 1ms for Δ*a* and *oc*. The proprietary software and the data set heatDS are available for academic studies by request.

### Features and feature sets

As features, we investigated the time series of raw input RR interval times (RR), together with the derived scalar features heart rate variability (RRvar) and average heart rate (RRmean); the HEAT optimal objective function value *F*(x*) (HEATobj) and the HEAT optimal solution (variable assignments) x* = (Δa*, bt*, oc*) (HEATsol).

Increasing accuracy and stability, we applied a moving horizon strategy to generate additional features. From the *n*_RR_ = 22 time intervals, we considered only $n_{\text{sub}}\in I≔\left\{10,\ldots ,n_{\text{RR}}\right\}$ on windows [1, 2, …, *n*_sub_] until [*n*_RR_ − *n*_sub_ + 1, 2, …, *n*_RR_]. This results in additional solutions $F_{i,n_{\text{sub}}}\left(x_{i,n_{\text{sub}}}^{*}\right)$ for i∈I. Investigating the robustness of solutions, we evaluated $F_{i,j}\left(x_{i,k}^{*}\right)$ for j,k∈I, the performance of the optimal solutions on time window *j* on time window *k*. We computed the features HEATobj and HEATsol for each subwindow of RR intervals. The moving horizon approach enabled us to compare of the HEAT simulation based on one time window with the raw RR intervals of a different one, as described above. We refer to the resulting time series of *n*_RR_ − *n*_sub_ + 1 entries HEATobj, HEATsol, and HEATfit as HEATseries, to the generically derived features mean and standard deviation as HEATseriesAvg. Finally, we also considered patient age (age). Table 1 summarizes the sets of features and resulting dimensions.

**Table 1:** Number of optimization parameters (pars), scaling factors, and hyperparameters (hyp) for the different feature sets and ML models.

Feature Set	included **Features**			
	ML Model	# Pars	# Scalings	# Hyp
rawRR	= {RR }			
	CNN	287–487	0	2
	SVM N-Gram	101–485	200–968	4
heatObjective	= {HEATobj }			
	SVM	2	2	4
heatSolution	= {HEATobj, HEATsol, RRvar, RRmean }			
	SVM	10	18	4
heatSerAvg	= {HEATseriesAvg }			
	SVM	21	40	4
heatSerAvgAge	= {HEATseriesAvg, age }			
	SVM	23	44	4
heatSeries	= {HEATseries }			
	SVM N-Gram	91–1691	180–3380	4

### Machine learning models

We used two classes of standard ML classification models: SVM and convolutional neural networks (CNN).

As SVM does not incorporate the temporal connection between sequential data, we first computed general features based on subsequences (N-Gram s) of the underlying data. These general features are the mean and the standard deviation of a given subsequence. For the mean, any subsequence with length ≥1 and ≤*n*_RR_ was considered. The standard deviation was only computed on subsequences of length ≥2. The hyperparameter *n*_sub_ limits the length of the time series before computing the features. Prior to training use, each feature was standardized to zero mean and unit standard deviation. The necessary parameters for this transformation were computed on the training set and used for the model evaluation. Based on these features, we implemented a SVM model in scikit-learn based on the LIBSVM library [61]. The underlying model is described in Cortes et al. (*“Support-vector networks”*) [62]. The kernel type (radial basis functions or polynomial) with a penalty parameter *C* and a kernel coefficient *γ* (3 values each) and the length of analyzed subsequences *n*_sub_ ∈ {10, …, 22} were tuned as hyperparameters using grid search cross-validation.

We used a CNN architecture consisting of two convolutional blocks followed by one fully connected layer with rectified linear unit (ReLU) activation functions and one final fully connected layer with a sigmoid activation function and output dimension one. Each of the convolutional blocks consisted of two convolutional layers with ReLU activation functions and five filters of width two followed by a max pooling and a dropout layer. The dropout rate (10%, 20%, 30%) and *n*_sub_ were tuned as hyperparameters during training using grid search cross-validation.

Other objective functions and architectures were evaluated manually in a preliminary phase, but eliminated as they gave no additional insight.

Table 1 shows the number of optimization parameters, scaling factors, and hyperparameters for the different approaches. The number of optimized parameters depends on the hyperparameter *n*_sub_ (the length of analyzed subsequences); therefore, ranges are provided. To avoid overfitting, each approach was evaluated on heatDS using repeated, stratified 10-fold cross validation to estimate performance on new data.

### Data

Our data set heatDS is a superset of one used in a previous study [17], which contains details of the data obtained from patients exhibiting AFib or AFlu with irregular ventricular response during invasive electrophysiological testing or catheter ablation. The retrospective data were extended to the period between 2011 and 2018 and 159 patients.

Classification AFib↔AFlu was performed using electrical signals measured at the atrial electrodes by an expert in the field of cardiac electrophysiology for all 159 patients. For AFib, we found that all examples exhibit highly irregular intervals of atrial activation (qualitative assessment) in combination with a short mean atrial cycle length (Δ*a*) of 182 ms. These data correspond well with the threshold of 200 ms, referenced in the European guideline for the management of AFib [63]. In contrast, intracardiac recordings taken from patients with AFlu exhibited highly regular intervals (Δ*a* ≈ 240 ms). In many cases, the correct rhythm diagnosis could be verified by evaluating the reaction of the arrhythmia to catheter ablation. Among the group of AFlu cases, further quantitative assessment revealed a Δ*a* variation below 5 ms.

We hypothesized that the dynamics of ventricular activations in short time periods contain enough information for successful discrimination. Therefore, we reduced the data complexity by extracting the time interval durations of 22 RR intervals from the surface ECG using built-in calipers, with a precision of 1 ms. Segments containing premature ventricular beats were excluded, which can be easily recognized by physicians or algorithms in clinical practice.

In summary, we collected 380 examples which were diagnosed either AFlu (*n* = 190) or AFib (*n* = 190). We used two or three disjoint examples per patient increasing the overall data size. We stored the time series of 22 values corresponding to RR intervals, the patient age, and the correct label AFib/AFlu for training and validation purposes. All other ECG data including the intracardiac measurements, were not considered with the exception of exemplary a-posteriori illustration. The study was approved by the University of Heidelberg Ethics Committee and conforms to the standards defined in the Helsinki Declaration.

In Kehrle (*“Inverse Simulation for Cardiac Arrhythmia”*) [59], we validated a previous version of our algorithm against other, smaller data sets from the publications focused on AFib↔AFlu discrimination. Unfortunately, there are no larger data sets available that can be used as an extended benchmark. Usually, these do not differentiate between AFib and AFlu specifically, or they do not classify supraventricular tachycardias at all, such as the American Heart Association ECG Database for example [64]. Therefore, all of the data in studies [8–11] could not be used, as it is unlabeled with respect to AFib↔AFlu.

### Implementation setting

All results were computed on a server running Ubuntu 16.04.4. The system had access to 1 TB RAM, an Intel(R) Xeon(R) CPU E5–2699A v4 at 2.40 GHz with 88 cores, and two NVIDIA(R) Quadro(R) p5000. The ML models were implemented using Python 3.5.2 and scikit-learn 0.20.3. The CNNs were based on tensorflow 1.8.0 and trained using the Adam optimizer [65] with default parameters. The computational times were roughly 20 ms per HEAT call (times 380 samples times number of considered subproblems per sample), 30 min for training SVM, and 3 d for training CNN.

## Results

### Accuracies for different feature sets and ML models

We show the mean accuracies and areas under receiver operating characteristic curves in Table 2. The results were obtained after repeated, stratified 10-fold cross validation for different feature sets and ML models as described in Sections Features and feature sets and Machine learning models.

**Table 2:** Average accuracies and areas under receiver operating characteristic (ROC) curve with standard deviations for the different approaches.

Feature Set	ML Model	Accuracy	ROC Area
rawRR	CNN	57.26% ± 6.47%	0.60 ± 0.08
	SVM N-Gram	62.03% ± 5.25%	0.66 ± 0.07
heatObjective	SVM	77.58% ± 4.15%	0.85 ± 0.05
heatSolution	SVM	79.37% ± 4.55%	0.87 ± 0.03
heatSerAvg	SVM	82.18% ± 4.48%	0.89 ± 0.03
heatSerAvgAge	SVM	82.47% ± 3.26%	0.90 ± 0.03
heatSeries	SVM N-Gram	82.84% ± 4.31%	0.90 ± 0.04

When directly applied to the input data of upto 22 RR interval times (rawRR), standard ML approaches achieved approximately 60%. The average accuracy increased to 77.58%, when $F_{i}\left(x_{i}^{*}\right)$ was used as the only feature (generated a priori from rawRR). A higher-dimensional classification, which also took $x_{i}^{*}$ and several HEAT solutions from a moving horizon strategy into account, increased the average accuracies to 79.37% and 82.84%, respectively. Using the best approach, we achieved a sensitivity of 87.21% and a specificity of 78.47%. An exemplary distribution of features is shown in Fig 6.

For an implementation of a CNN, the poor performance of direct application to rawRR was also reflected by high standard deviations. The number of ML parameters was two orders of magnitude larger than that for SVM, although only few layers were chosen due to the small size of the training set and compared to DL approaches to cardiac arrhythmia classification [3]. The SVM results were considerably stable and no significant differences occurred for different kernel types. The approach to pre-process rawRR using medical expert knowledge (HEAT) can be seen as an approach that increases sensitivity without overfitting the ML model.

### Interpretability

**Fig 4:**
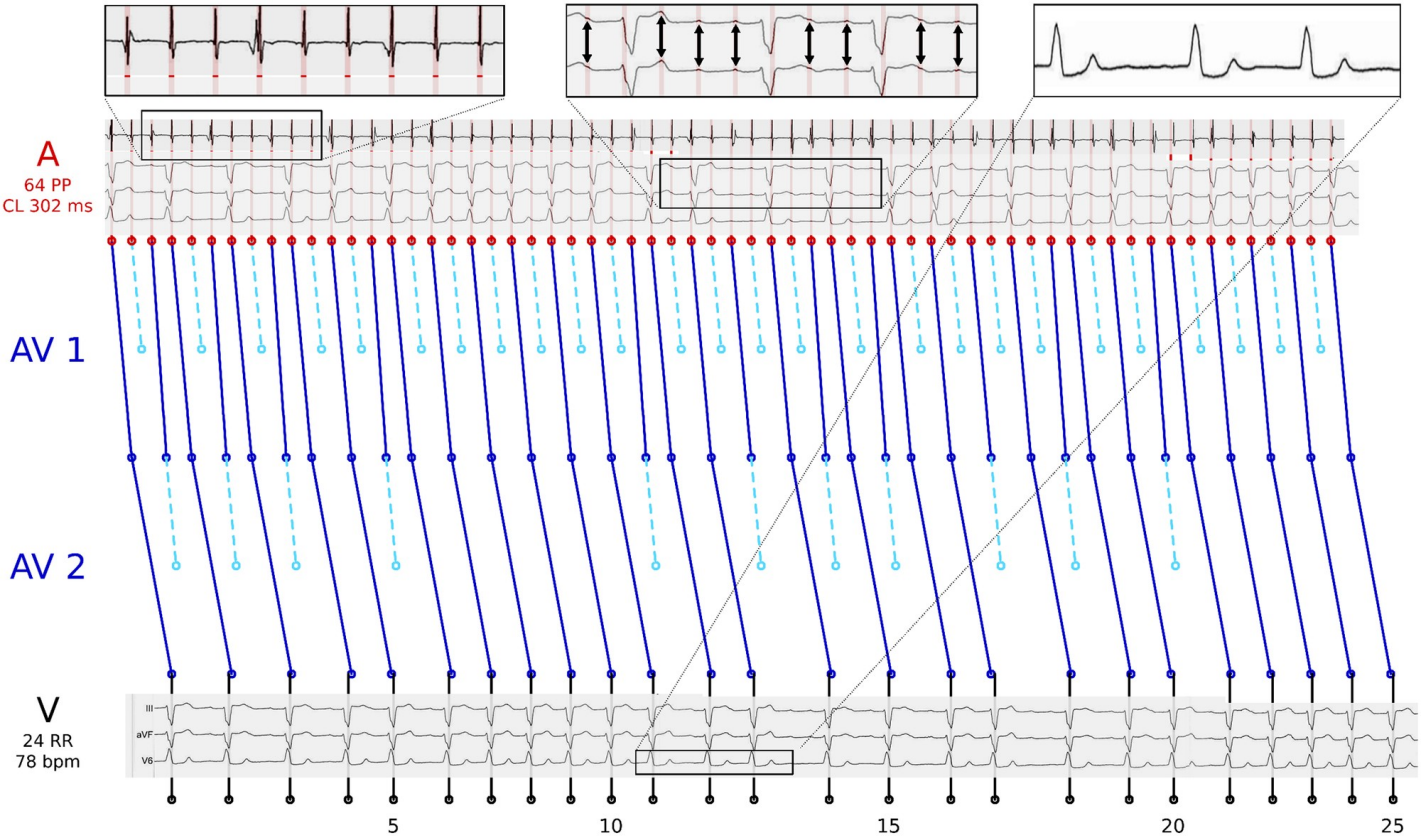
Exemplary illustration of how the feature *atrial cycle length* derived from a HEAT solution can be a posteriori pathophysiologically interpreted and used.

We observed that the calculated objective function values $F_{i}\left(x_{i}^{*}\right)$ were the most decisive feature for classification, and the features associated with $x_{i}^{*}$ are interesting for clinical interpretation. Fig 4 shows how knowing the atrial cycle length Δa* may be helpful for an a-posteriori identification of flutter waves for AFlu in a surface ECG. The figure shows observed and simulated data, as in left-hand side of Fig 2, but for different input data from the same patient. The actual atrial cycle length is only available with invasive procedures and is difficult to identify from investigating the surface electrocardiogram (ECG, rightmost zoom), where almost no atrial activation is recognizable. The intracardiac measurements are shown for illustrative purposes and coincide with the value Δ*a* proposed by HEAT (leftmost zoom). When no intracardiac measurements are available, this value Δ*a* can help the physician, when reanalyzing the ECG. An overlay of Δ*a* makes spotting atrial activations in the surface ECG easier (middle zoom).

**Fig 5:**
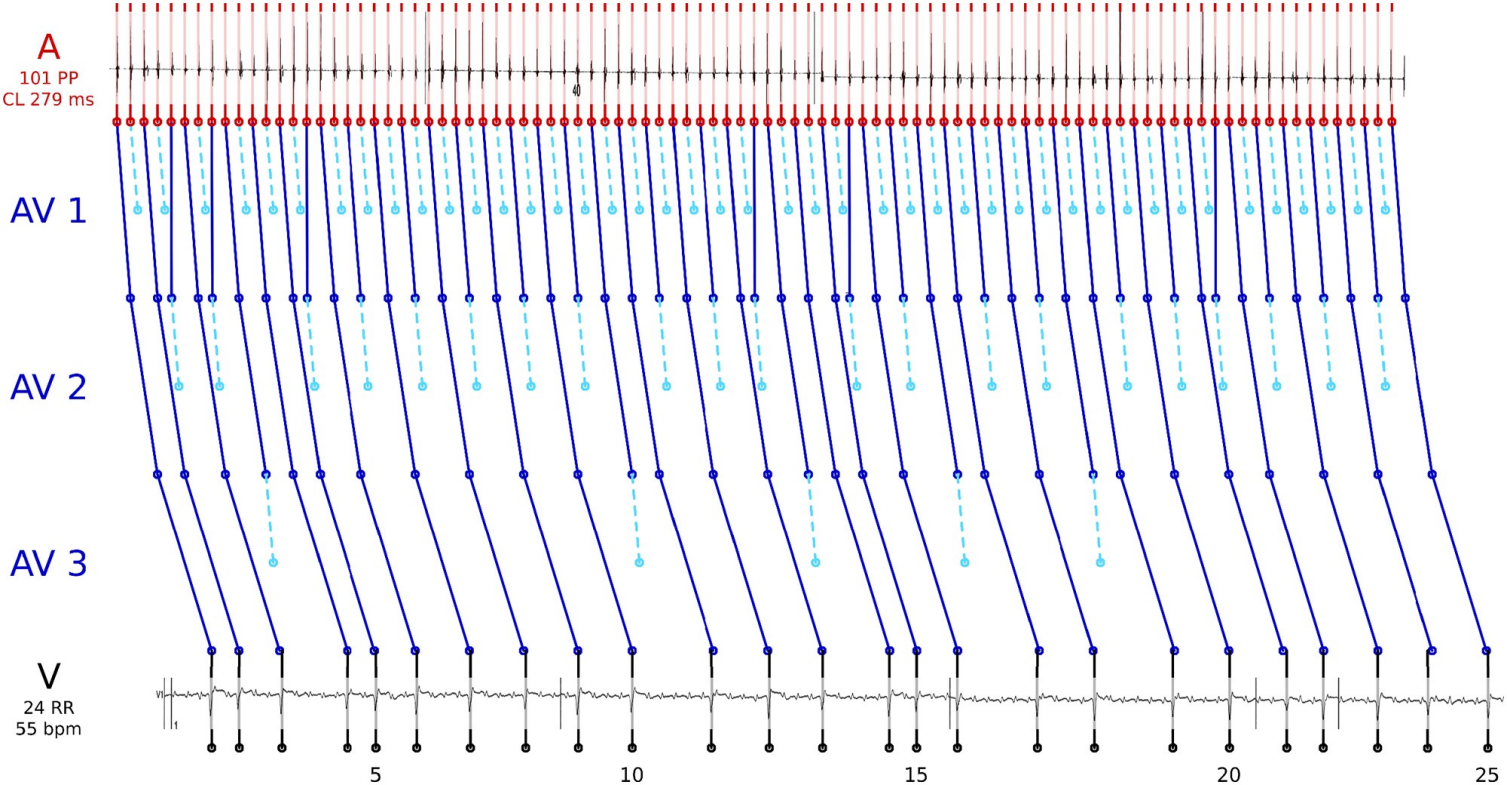
Exemplary illustration of how the feature *blocktype* derived from a HEAT solution can be a posteriori pathophysiologically interpreted and used.

Fig 5 shows observed and simulated data, but for different input data. Here, a three-level atrioventricular (AV) block with a varying 2:1 / 3:2 level followed by two levels with a varying 1:1 / 2:1 conduction was calculated (MAVB 3 in Fig 3). Again, the intracardiac measurements are shown for illustrative purposes (top). The close match to the calculated atrial cycle length Δ*a* highlights the plausibility of the complex blocking mechanism. The optimal blocktypes bt*, compare Figs 4 and 5 with two and three levels with varying blockings, respectively, give insight into the pathophysiology of the AV node and may be useful for treatment planning.

The high accuracy of ML approaches that used HEAT-generated features indicates that our novel mathematical model is an appropriate description of the complex blocking mechanism for AFlu.

### Moving horizon approach

The results in Table 2 seem to indicate that additional accuracy can be obtained using the feature HEATseries. It consists of time-series data generated from several calls to HEAT for input data obtained from a moving horizon approach. As explained above, *n*_sub_ ∈ {10, …, *n*_RR_} was optimized as a hyperparameter, with *n*_sub_ = 17 giving the best results. The overall number of time intervals *n*_RR_ = 22 was fixed. Therefore, the time series in HEATseries corresponded to entries for six different optimization problems (1 …17 to 6 …22).

An interesting and promising question is regarding how much the approach can be improved for larger values of *n*_RR_. Unfortunately, the idea to use several optimization results in one feature set was presented after data from many patients with small numbers of RR intervals were already collected. Considering the collected number of RR intervals for the 159 patients, the average number is 51 with a range from 22 to 111. This made a rigorous cross-validated comparison of larger values of *n*_RR_ difficult as our data base was simply not large enough. A study showed large potential with accuracy increasing from 82.94% to 92.50% for long time horizons of *n*_RR_ = 90 intervals. However, this result needs to be cross-validated on larger data sets.

## Discussion

### Impact, accuracy, and applicability

Being able to classify AFib↔AFlu is clinically relevant. There are a variety of treatments (antiarrhythmics, various ablations and ablation systems) with different side effects and cure rates. A correct classification is imperative to choose the best treatment [15]. Therefore, use of the proposed approach for clinical decision support may be helpful, especially when considering the excellent classification accuracy and interpretability of calculated features and the difficulty of the classification task for unexperienced clinicians.

All ML approaches that were applied directly to the input data (rawRR) resulted in average accuracies of approximately 60%. These low accuracies were not surprising, as AFib↔AFlu is a difficult case even for experts [12–14] and was explicitly excluded in recent studies [3]. AFib may be overdiagnosed because of coarse fibrillatory waves, which are reminiscent of AFlu [13, 66], the presence of artifacts, or premature atrial complexes [67]. AFlu may be overdiagnosed because the low-voltage flutter waves that indicate AFib are barely discernible in the surface ECG (compare Figs 2 and 4), or because a pseudo-regularization may occur [68] (see Section Classification failures). The achieved accuracies are similar to previous results to analyze AFib↔AFlu, e.g., based on clustering of RR times or nodal recovery approaches [59]. Note that the N-Gram approach implicitly considers RRvar, RRmean and is thus a superset of features used in current smartwatch algorithms [8]. Hence, the low accuracy gives a hint why AFib↔AFlu is currently untreated by them.

Using HEAT for an a-priori calculation of heatObjective was significantly more successful with an average accuracy of 77.58%, even though the input data was identical (rawRR). Using heatSolution features resulted in an increased average accuracy of 82.84% (sensitivity 87.21%). Further improvements can be expected if settings of the HEAT algorithm (such as a lower bound on Δ*a* or grid sizes) were optimized as hyperparameters, if underlying model assumptions were adapted after careful analysis of wrongly classified samples, once more training samples become available, and if covariates were considered. Age (heatSerAvgAge) did not seem to have a significant impact on accuracy.

Using ML with HEAT-generated features has the drawback; Each classification sample requires calculating the optimal solution of the MAVB. However, the additional 20 ms should be acceptable in a clinical context and negated by several advantages:

First, the approach is applicable in clinical practice. We assumed in a previous assessment that the presence of either AFib or AFlu was verified. A different perspective shows, our approach is a reasonable complement to generic DL approaches for cardiac arrhythmias [3]. This can use the prior one-cluster classification of AFib and AFlu, and can classify AFib↔AFlu in a following step. HEAT can run on a secure client-server, which was implemented by [59]. It can communicate with a smartphone app that generates rawRR data from ECG-derived pictures or beeps from a heart monitor. A similar procedure can be implemented for wearables and smartwatches.

**Fig 6:**
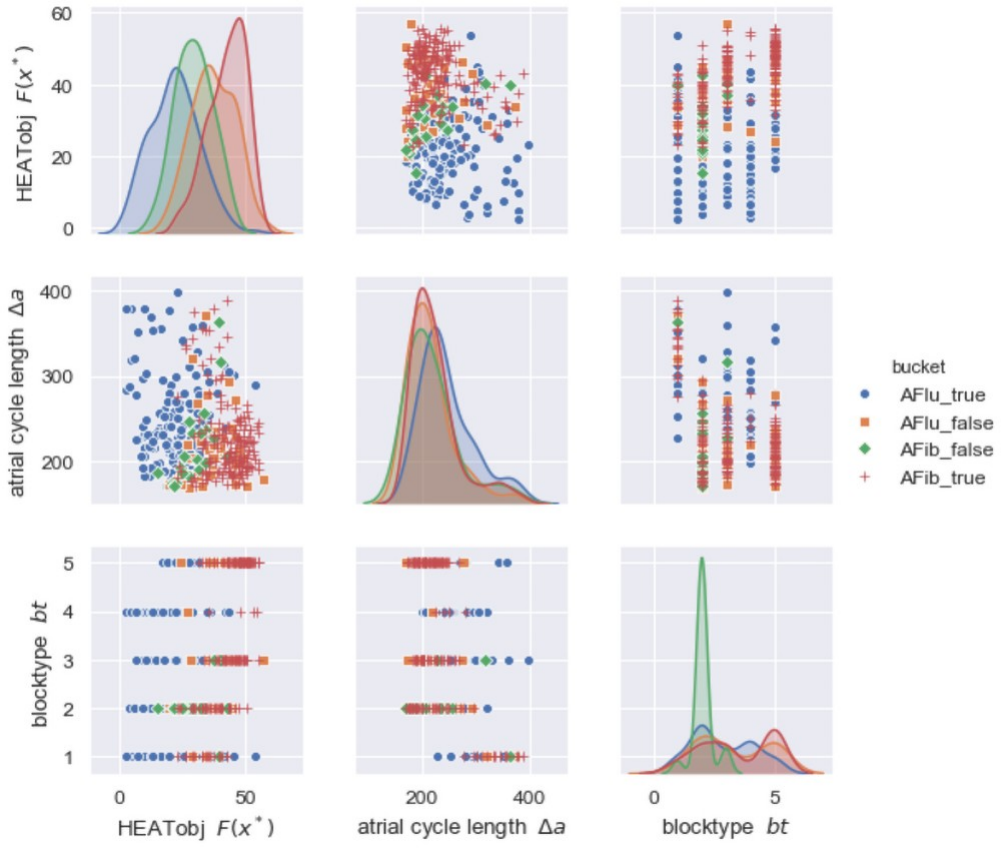
Representative pairwise plot of features obtained from a heatSolution SVM classification, compare Table 2.

Second, the dominance of the HEATobj feature and the availability of a distribution (compare Fig 6), allow calculation of a probability for the classification (the higher the value, the more likely AFib). Such a value would help clinicians determine the validity of a suggested diagnosis. In Fig 6 the clear separation of atrial flutter (AFlu) and atrial fibrillation (AFib) with respect to HEATobj is observed. The two model parameters in x*, the atrial cycle length Δ*a* and the blocktype *bt*, do not allow a straightforward classification.

Third, the approach results in a high accuracy. It is an open question whether a similar accuracy can be achieved with DL without the explicit modeling of expert knowledge. Probably yes, if the number of verified training samples, hidden layers, and computational resources is large enough. However, the approach would lack interpretability.

### Interpretability

Interpretability is the fourth and most important advantage of the proposed approach.

We reduced the complexity of the data a-priori by considering only time points of the clearly visible R waves (the beeps of a heart rate monitor) corresponding to ventricular activation. This makes the underlying data more assessible to humans. HEAT provides HEATsol, the optimal solution x* = (Δa*, bt*, oc*). These values can be interpreted by experts, and used for maiking treatment decisions. For example, the atrial cycle length Δa* proposed by HEAT can help the physicians when reanalyzing the ECG (compare Fig 4). Furthermore, the absolute cycle length can help identifying patients with typical atrial flutter (Δ*a* ∼ 200 ms) or predicting procedural success [7]. In addition, for AFlu *a thorough understanding of electrophysiological properties and anatomical landmarks is essential in achieving a successful ablation outcome and in reducing complication rates* [69]. Sometimes it is even claimed that *the classic ECG-based diagnoses of tachycardias and AFib are of little importance today because treatment is based on the direct management of the trigger mechanism* [70]. We believe that estimates of the atrial cycle length or the blocktype (compare Figs 4 and 5) can be a valuable asset to clinical decision making.

### Impact of ML architectures and feature selection on accuracy

Table 2 shows the accuracies for different machine learning architectures. After reasonable effort to investigate different architectures, none resulted in an accuracy significantly above 60% when directly working with rawRR. We think that this is mainly due to the comparatively small amount of data samples and the difficulty to tailor standard ML architectures to the specific time series character of RR intervals. When the features that were generated using domain knowledge were considered, SVM outperformed our CNN architectures as discussed in the next subsection. We expect a different behavior if neural network architectures are used that explicitly address time series, such as recurrent networks.

A key ingredient in the proposed approach is the generation of features via domain knowledge. We solved an inverse optimization problem for the mathematical MAVB model introduced in Section Multilevel atrioventricular block (MAVB). This generic approach is preferable for the aforementioned reason of interpretability and it obsoletes the cumbersome tailoring of a generic neural network architecture for the specific classification task obsolete. The classification in the low-dimensional feature space can be efficiently and accurately done with SVMs.

The selection of features was straightforward, as there are only a few model parameters that are calculated along with the objective function value. The latter alone was decisive and was enough for a high-accuracy one-dimensional linear classifier, using a simple threshold value compare the entry for heatObjective in Table 2. The additional features considered in heatSolution increased accuracy, although we see the main benefit of block type, atrial cycle length, and conduction constants in the physiological interpretability. Future work should focus on consideration of sets of optimal solutions and solutions on moving time horizons. In this context, the impact of heatSolution may improve.

### Approximation properties of ML approaches

**Fig 7:**
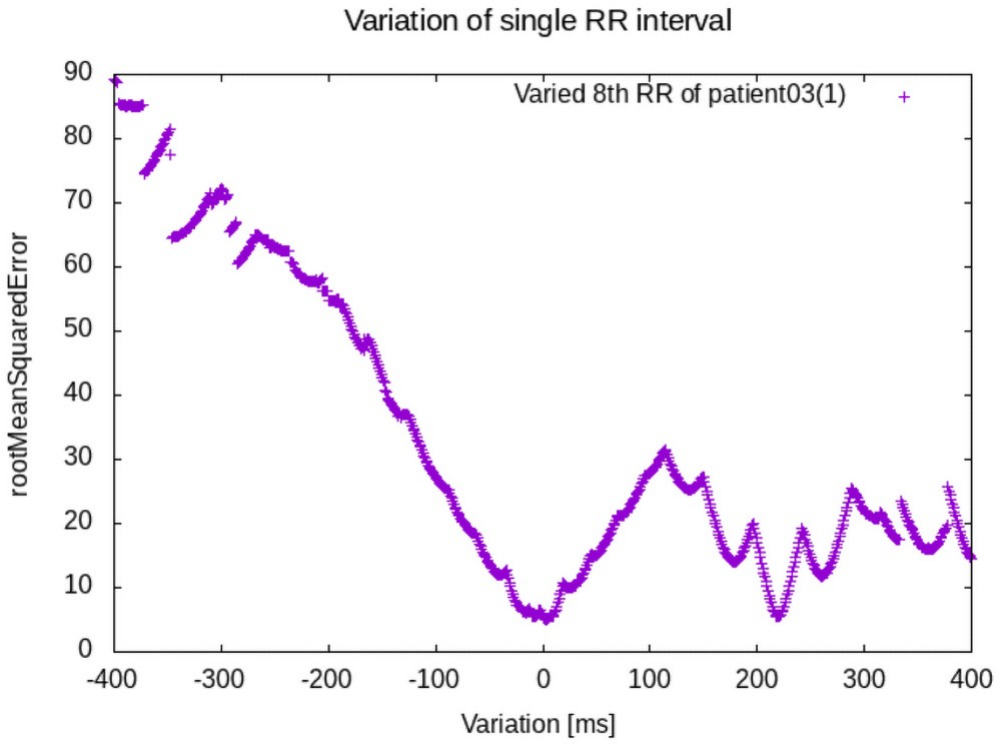
Fluctuation of the objective function of our mathematical model with respect to shifts in one input signal of a data sample.

It is well known that feed-forward neural networks are universal approximators of continuous functions, if either the number of neurons on one hidden layer [71] or the number of layers for a fixed number of neurons per layer [72] increase. However, it is also well known that these theoretical results are obtained at the price of a potentially large number of weights distributed over the hidden layers of the neural net. Adaptive activation functions have better approximation properties [73], but the main difficulty of current architectures is the same. To get an idea why CNNs do not perform well on AFib↔AFlu, for deep nets with 34 layers as in [3] as well as in our prototypical implementation, we analyze Fig 7.

Fig 7 shows the feature HEATobj, the optimal objective function value *F*_*i*_(*x*) provided by HEAT, for 801 different artificial input vectors *x*. As input, 17 RR intervals of an exemplary patient were chosen. Sixteen of them are kept fixed, while one particular interval length in the middle was varied with deviations of -400 ms to +400 ms in intervals of 1 ms. The plot shows locally quadratic behavior, due to the quadratic objective function (Euclidean norm). The discontinuities are due to the clipping of solutions that result in deviations of more than 150 ms between signals. The main takeaway from the plot is that the minimal objective function value as a function of the input consists of many piecewise quadratic segments. Estimating the number of ReLU-induced linear segments necessary to approximate this important feature for classification, one easily reaches large numbers: assume 20 linear segments, and use *n*_sub_ = 17 as an exponent. Of course, the feature HEATobj is only an approximation of the real process, but the mathematical modeling based on physiological knowledge and the high accuracy indicate that the real MAVB will show a similar behavior. Given the additional difficulty for this classification task, only a few labeled training data sets are available. We conjecture that it will be difficult to train CNNs with a reasonable classification accuracy without using domain knowledge.

### Classification failures

**Fig 8:**
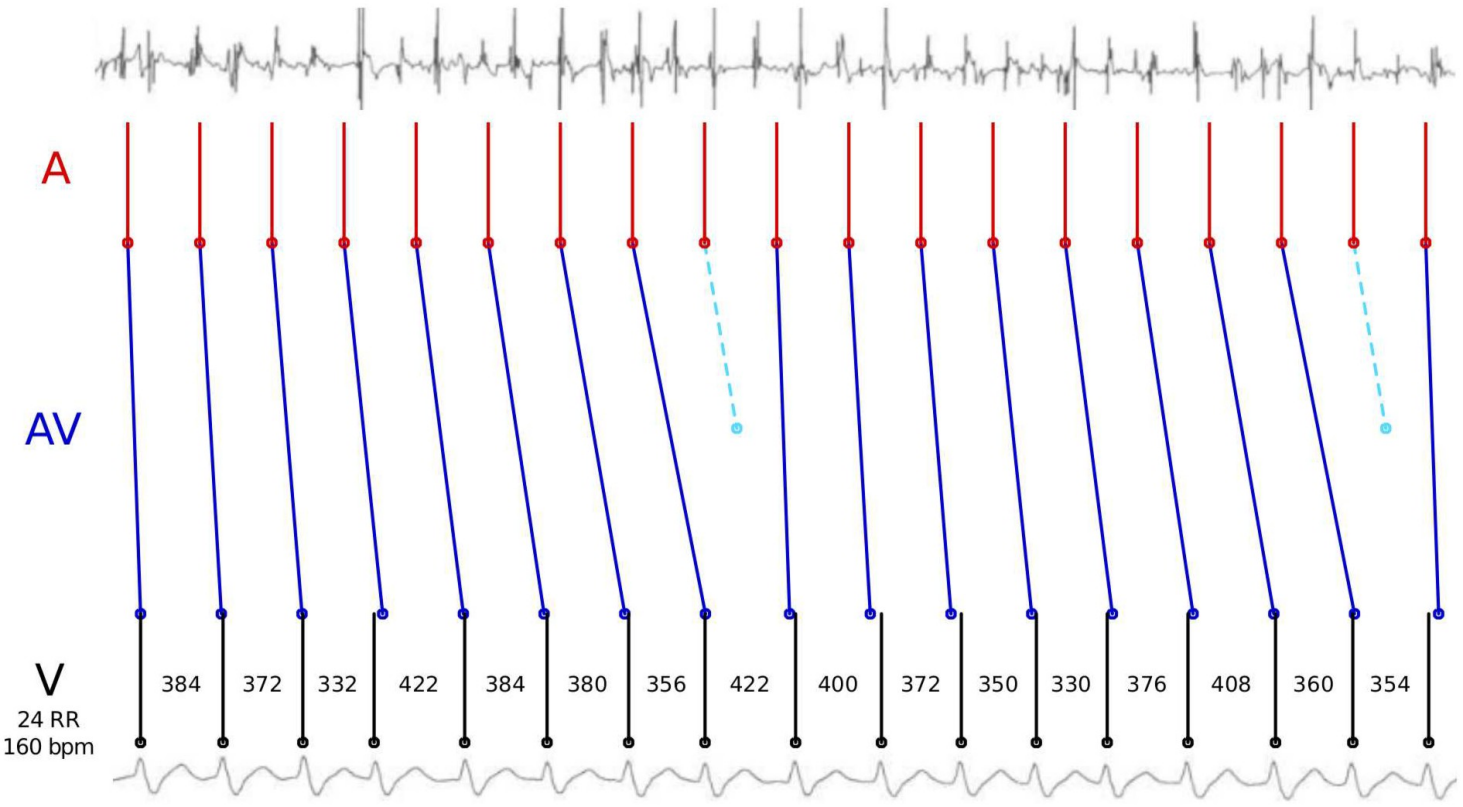
Example of an atrial fibrillation input that is misclassified due to pseudo-regularization.

While our novel approach resulted in excellent area under the curve values, there were still misclassification samples. Fig 8 shows an atrial fibrillation case with a very fast (160 beats per minute), but pseudo-regular ventricular contraction, shown in the surface lead at the bottom. The atrial contraction, however, is totally chaotic as shown by intracardiac measurements displayed in the top. Due to this pseudo-regularization, the best MAVB simulation matched the observed data considerably well and led to a misclassification. It is well known that at very high frequencies of AFib, a pseudo-regularization can occur [68]. Here, the RR variability decreases with an increase in heart rate, which leads to an almost regular rhythm despite a totally chaotic atrial contraction. As a consequence, these AFib cases with high ventricular rates may be more likely to match a regular MAVB or even a 1: 1 conduction. In our approach, pseudo-regularizations result in relatively low objective function values which impair correct classification.

Just as for experts, the presence of artifacts or premature atrial complexes [67] may lead to a misclassification. It is an open question how to extend the mathematical model in Section Multilevel atrioventricular block (MAVB) for automatic detection of pseudo-regularization and increased specifity without impairing sensitivity. Using the feature *atrial cycle length* more elaborately or additionally classifying the flutter waves may be helpful in this context.

An intrinsic limitation for classification accuracy using our approach arises from false positives, cases of AFib that “by chance” are very close to multilevel blocks. The mathematical question of how dense random rawRR instances are in the space of all MAVB solutions is open.

### Generalization to other cases of clinical decision support

Our proposed approach can be generalized as *enhance ML approaches by features based on understandable and interpretable mathematical models of clinical expert knowledge that exhibit complex dynamic behavior*. Personalizing these mathematical models results in model parameters that can be used for classification, prediction and dynamic stratification, but also be interpreted by clinicians. Diagnosis of other cardiac arrhythmias could be done in a similar way. For diseases such as acute leukemias [74, 75] or polycythemia vera [76], there are mathematical models that have been validated with measurement data, and contain estimated personalized model parameters like stem cell proliferation rates. Such hidden parameters usually cannot be observed directly and can be very useful for clinical decision-making [60].

Our interdisciplinary approach with cardiologists and mathematical optimizers has several obvious benefits [77]. One of them is that the role of HEAT can be seen as a well-informed agent interacting with a surrounding machine learning environment. Such an approach was introduced and discussed in Holzinger (*“Interactive machine learning for health informatics: when do we need the human-in-the-loop?”*) [78]. The paper exactly emphasizes the benefits of human expertise and the search for unknown patterns in a low-dimensional feature space upon which our approach is based.

We believe that it is better to use interpretable models than to explain black-box models [79]. An integration of interpretable expert systems written as optimization models with today’s powerful ML approaches may result in better healthcare with interpretable results.

## Conclusions

We proposed a method for the difficult classification task AFib↔AFlu that combines expert models and ML. On our gold standard test set, our approach was highly successful reaching a classification accuracy of 82.84% and area under the ROC curve of 0.9. In contrast, for short RR time series and comparably few labeled training samples, we could not achieve such an accuracy with a purely data-driven ML model.

Our work ideally complements deep-learning-based methods, which can provide a pre-classification, but cannot distinguish between AFib and AFlu. However, this distinction is highly relevant from a clinical perspective. The classification itself, together with corresponding features calculated by HEAT, may be interpreted by medical experts and used for determing treatments. As runtimes of the algorithm are short enough for real-time requirements, it can be applied as a decision-support tool for clinical practice. A combination of the presented feature extraction and classification with state-of-the-art NN is plausible, but open due to availability of data sets and trained models. An open question is how to further reduce failure cases due to pseudo-regularization as discussed in subsection Classification failures.

Finally, we proposed to create features from optimal solutions of domain-knowledge models and to search for unknown patterns in a low-dimensional feature space. We think this general approach of combining the interpretability of expert systems with the deductive power of data-driven ML can and should be transferred to other cases of clinical decision support.

## Supporting information

S1 File (ZIP)
